# HIV Prevalence Trends, Risky Behaviours, and Governmental and Community Responses to the Epidemic among Men Who Have Sex with Men in China

**DOI:** 10.1155/2014/607261

**Published:** 2014-04-14

**Authors:** Eric P. F. Chow, Joseph T. F. Lau, Xun Zhuang, Xiaohu Zhang, Yanjie Wang, Lei Zhang

**Affiliations:** ^1^The Kirby Institute, Faculty of Medicine, University of New South Wales, Sydney, NSW 2052, Australia; ^2^Central Clinical School, Faculty of Medicine, Nursing and Health Sciences, Monash University, Melbourne, VIC 3800, Australia; ^3^Melbourne Sexual Health Centre, Alfred Health, Carlton, VIC 3053, Australia; ^4^Comprehensive AIDS Research Center, School of Medicine, Tsinghua University, Beijing 100084, China; ^5^Centre for Health Behaviors Research, School of Public Health and Primary Care, Faculty of Medicine, The Chinese University of Hong Kong, Hong Kong Special Administrative Region, China; ^6^School of Public Health, Nantong University, Nantong, Jiangsu 226019, China; ^7^Department of Sociology, Tsinghua University, Beijing 100084, China; ^8^China Food and Drug Administration Institute of Executive Development, Beijing 100073, China

## Abstract

*Purpose of Review.* Numerous studies reported the rapid spread of HIV/AIDS epidemic among men who have sex with men (MSM) in China. This paper aims to investigate the overall epidemic trend and associated high-risk behaviours among Chinese MSM and to explore the governmental and community responses to the epidemic. *Recent Findings.* HIV prevalence among Chinese MSM increased rapidly in all Chinese regions in the past decade and disproportionally affected the Southwest China. In addition to the high-risk homosexual behaviours, overlapping bisexual, commercial, and drug use behaviours are commonly observed among Chinese MSM. The Chinese government has significantly expanded the surveillance efforts among MSM over the past decade. Community responses against HIV have been substantially strengthened with the support of international aid. However, lack of enabling legal and financial environment undermines the role of community-based organisations (CBOs) in HIV surveillance and prevention. *Conclusion.* HIV continues to spread rapidly among MSM in China. The hidden nature of MSM and the overlapping homosexual, bisexual, and commercial behaviours remain a challenge for HIV prevention among MSM. Strong collaboration between the government and CBOs and innovative intervention approaches are essential for effective HIV surveillance and prevention among MSM in China.

## 1. HIV Epidemic in China 

Estimated 780,000 people in China are currently living with HIV/AIDS, accounting for 0.057% of the Chinese population in 2011 [1]. HIV epidemic was initiated and mainly transmitted by sharing injecting equipment among injecting drug users (IDU) in China in the past decade [2, 3]. However, sexual transmission, especially male-to-male homosexual transmission, has become the major mode of HIV transmission in recent years [4, 5]. The latest national report revealed that the proportion of newly diagnosed HIV cases due to male homosexual contact has increased from 12.2% in 2007 to 32.5% in 2009 [5]; while the national HIV prevalence among MSM had a 4.5-fold increase in the past ten years (i.e., from 1.4% in 2001 to 6.3% in 2011) [1, 6]. The level of HIV prevalence in MSM is still relatively low compared to other Asian countries such as Cambodia (7.8%), Indonesia (9.0%), and Thailand (24.6%) [7]. Currently, Chinese MSM represents about 2–4% of the sexually-active male population in China (i.e., 5–10 million) [8, 9].

The rapid spread of HIV epidemic among Chinese MSM has become a national concern [10]. Several published review articles on Chinese MSM have described the HIV disease burden and its transmission through high-risk sexual behaviours among MSM in China [11–13]. Extended from previous findings, this paper aims to (1) describe the trend of HIV epidemic among MSM in relation to the less investigated overlapping risk behaviours and (2) assess the current health polices and surveillance efforts from the Chinese government and community responses to the epidemic. Assessing these specific aspects provides insightful implications for the country's public health responses and informs relevant health policies.

## 2. Rapid Transmission of HIV among Chinese MSM

**Figure 1:**
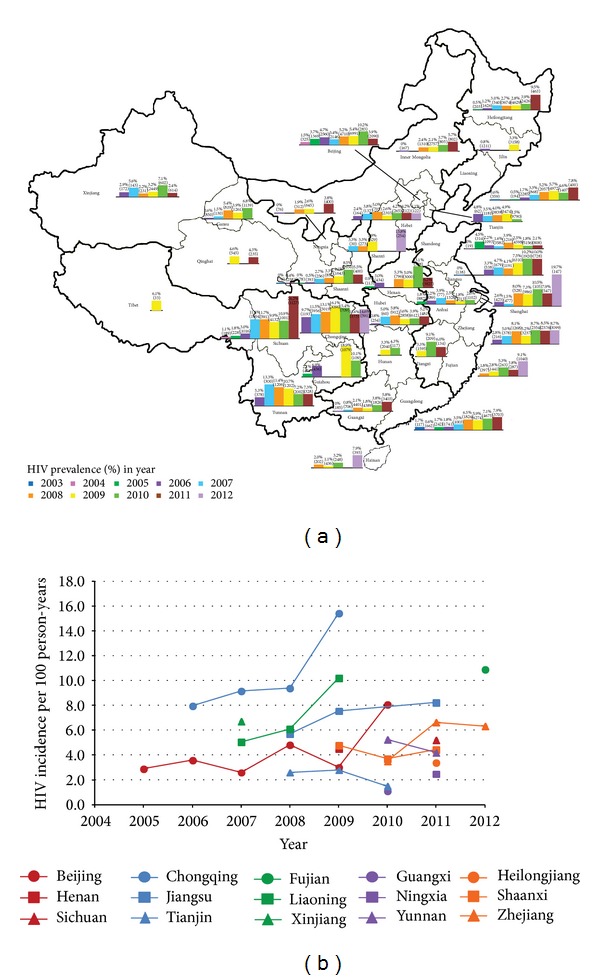
(a) HIV prevalence among MSM in China (2003–2012). HIV prevalence in Anhui [48–57]; Beijing [48, 58–81]; Chongqing [20, 35, 36, 48, 82–92]; Fujian [48, 93–98]; Gansu [36, 48, 99–101]; Guangdong [17, 46, 48, 52, 102–124]; Guangxi [48, 125–138]; Guizhou [48, 139–144]; Hainan [48, 68, 145–147]; Hebei [48, 148–157]; Heilongjiang [20, 48, 54, 69, 76, 77, 100, 158–166]; Henan [20, 48, 54, 69, 76, 77, 167–174]; Hubei [20, 48, 63, 175–179]; Hunan [48, 180–182]; Inner Mongolia [48, 100, 183–187]; Jiangsu [20, 48, 68, 188–206]; Jiangxi [48, 207–210]; Jilin [48, 100]; Liaoning [20, 48, 55, 68, 100, 163, 211–223]; Ningxia [48, 100, 224, 225]; Qinghai [226, 227]; Shaanxi [20, 48, 68, 228–231]; Shandong [48, 68, 180, 232–247]; Shanghai [20, 48, 68, 248–252]; Shanxi [48, 253–255]; Sichuan [20, 48, 69, 76, 77, 123, 256–268]; Tianjin [48, 68, 269–274]; Tibet [48, 275]; Xinjiang [48, 276–281]; Yunnan [48, 68, 168, 282–290]; and Zhejiang [48, 68, 291–303]. The percentages on the bar chart represent the prevalence of HIV infection among MSM and the numbers in the round bracket represent the total number of MSM screened; (b) HIV incidence among MSM in China (2005–2012). HIV incidence in Beijing [64, 70, 74, 304, 305]; Chongqing [306–308]; Fujian [98]; Guangxi [309]; Heilongjiang [310]; Henan [311]; Jiangsu [190, 312–316]; Liaoning [55, 216]; Ningxia [317]; Shaanxi [230, 318]; Sichuan [319]; Tianjin [272, 274]; Xinjiang [279]; Yunnan [289, 320]; and Zhejiang [299, 321].

Growing trends of HIV epidemic among MSM have been observed in all Chinese provinces, municipalities, and autonomous regions; however, the epidemic varies geographically and temporally (Figure 1(a)). The national HIV prevalence has increased rapidly from 0.9% in 2003 to 6.3% in 2011 [14]. Several studies have also indicated that MSM recruited from gay venues (i.e., gay bars, saunas, and bathhouses) have a higher HIV prevalence compared with those MSM recruited from the Internet, clinics, or other settings [15–17]. Furthermore, HIV incidence have also increased in several municipalities and provinces (Figure 1(b)), such as Beijing Municipality (from 2.9 to 8.1 per 100 person-years during 2005–2010), Chongqing Municipality (from 8.0 to 15.4 per 100 person-years during 2006–2009), Liaoning Province (from 5.1 to 10.2 per 100 person-years during 2007–2009), Jiangsu Province (from 5.7 to 8.2 per 100 person-years during 2008–2011), and Zhejiang Province (from 3.5 to 6.3 per 100 person-years during 2010–2012). Provinces in the Southwest and Northwest regions consistently exhibit higher prevalence levels than other parts of China. Previous study showed that the proportion of all reported diagnosed cases that were attributed to male homosexual exposure in the city of Kunming of Southwest China has increased from 2.2% in 2000 to 42.2% in 2007 [18].

## 3. High-Risk Homosexual Behaviours 

Approximately 85% and 90% of MSM have had anal sex and oral sex with men in the past six months, respectively [19, 20], and each Chinese MSM has approximately 7.2 ± 17.3 oral sex partners and 6.6 ± 15.6 anal sex partners [20]. Correct and consistent use of condoms can prevent HIV transmission by 85–90% [21–25]; however, condom usage varies across types of sexual partnerships among Chinese MSM. A recent systematic review and meta-analysis have demonstrated that regular male partnerships in Chinese MSM have the lowest consistent condom use during anal intercourse over the past six months (19.9%) compared with noncommercial casual (30.4%) and commercial partnerships (58.0%) [26]. Low condom use is mainly due to the preference of better sexual sensation and the fear of making partners feeling untrusted. Since most of the MSM perceive oral sex as a ‘‘safe-sex” activity [27], the rate of condom use in oral sex is extremely low (~10%) [20, 28]. Apart from these, a substantial proportion of MSM also have participated in other sexual contacts such as rimming (26.0%) and fisting (27.6%) [20]. About 27.7% have reported experiencing bleeding during or after intercourse [20]. Although these unusual sexual practices are considered as low risk sexual activities, the exchange of body fluids (i.e. blood and semen) could possibly facilitate HIV transmission [29]. In addition, nonmonogamous relationships are common among Chinese MSM [14, 30, 31]. National behavioural surveillance reported that the proportion of MSM who had multiple male sex partners in the past six months increased from 68.0% in 2008 to 85.4% in 2011 [14], and about 18.6% have participated in group sex activities in the past 12 months [20].

## 4. Prevalent Overlapping Risk Behaviours

### 4.1. Bisexual Behaviours

‘‘Of the three kinds of unfilial conducts, having no posterity to continue the family line is the gravest” is one of the traditional family values in China [32]. Chinese parents expect children to marry and have children to continue the family line [33, 34]. Previous studies have reported that 25–35% of Chinese MSM are currently married to a female [35–37] and over 70% of MSM will potentially enter a heterosexual marriage during their lifetime due to social and family pressure [38–40]. Married MSM often have unprotected sex with their wives not only for the reason of reproduction [41], but also an indication of husbands' fidelity to their wives [42]. It has been reported that the rate of consistent condom use between MSM and female partners in the past six months is only 23.3% (95% CI: 11.3–42.1%) [12]. Moreover, a systematic review and meta-analysis estimated that about 68.0% of the HIV-positive Chinese MSM have unprotected vaginal intercourse (UVI) with their female partners [43]. Given that MSM who have sex with women is 1.3 (95% CI: 1.0–1.6) times higher risk of HIV compared to MSM who have sex with men only; female partners of bisexual MSM are at higher risk of HIV [44]. A recent study estimated that the HIV incidence among female partners of bisexual MSM has significantly increased 5.3 fold from 0.18 per 1000 person-years in 2002 to 0.88 per 1000 person-years in 2010 in China [45]. Bisexual behaviours of MSM pose potential threats of bridging HIV transmission to their female partners, spreading the epidemic into the general female population [46]. Disclosure of homosexuality is not common and only 11% of married Chinese MSM have disclosed their homosexuality to their wives [39]. Sexual harassment is common between homosexual men and their female partners, and 30% of wives have reported experiencing domestic violence by their homosexual or bisexual husbands [47].

### 4.2. Commercial Sexual Activities

Available studies have suggested that substantial proportion of Chinese MSM is also involved in the male-to-male commercial sex trade. Approximately 6.5–22.6% of the Chinese MSM have paid for sex with men [58, 232, 322, 323]; on the other hand, about 4.9–24.3% have sold sex to men in the past six months [58, 322–326]. In addition, male sex workers are coined as “money boys” or informally as “*yāzǐ*” (duck; who serves male and female clients) and “*é*” (goose; who serves male clients only) in the Chinese context [327]. Money boys have borne a disproportionate burden of HIV infection [11, 328–331]; however, very little epidemiological and sociobehavioural studies focus on this subpopulation. Previous studies have shown that money boys are usually younger, less educated, and more likely to have unprotected sex with multiple male clients compared to the broader MSM population [11, 328–330]. A survey has reported that about 13.2% of MSM are engaged in paid sex activity but only 59.7% have used condom at every anal sex over the past six months. Additionally, nearly half (i.e., 43.1%) of the money boys also have a heterosexual partnership but only 36.0% use condom with their female partners over the past six months [329]. Most of the money boys often move between cities for sex trade in order to avoid being recognised by the local community [11, 19, 328], such domestic migration potentially facilitates the transmission of HIV across geographical locations [332–334]. Recent review demonstrated that the odds of exposure to HIV among money boys are 1.3 (95% CI: 1.1–1.5) times higher than the odds of exposure to HIV among the broader MSM population [11].

### 4.3. Injecting Behaviours

It is shown that about 8% of MSM who also have injected drugs in the past 12 months [20]. China has a long history of illicit drug trafficking and high rates of HIV infection among IDU [2, 3, 335]. Recent national report revealed that six out of the 31 Chinese provinces (i.e., Yunnan, Xinjiang, Guangdong, Guangxi, Sichuan, and Guizhou) accounted for 84.2% of the HIV epidemic among the IDU population [336]. At the same time, MSM in these provinces also have the highest HIV prevalence across the country (Figure 1(a)). The overlapping risk behaviours among MSM who also inject drugs (MSM-IDU) are likely to facilitate HIV transmission [337, 338]. Currently, the injecting and needle-sharing behaviour among MSM in China is little known. It remains a challenge to promote public health interventions to this overlapping population [339].

## 5. Governmental Responses to HIV among Chinese MSM

**Figure 2:**
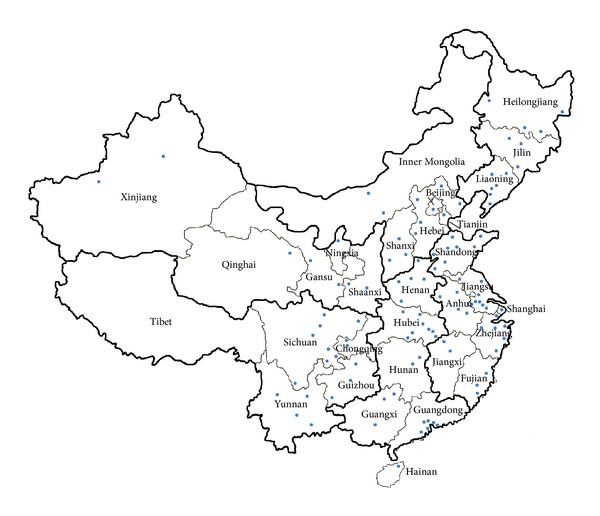
Distribution of HIV national sentinel surveillance sites for men who have sex with men in China. The blue dots represent the location of 109 sentinel surveillance sites in 2011.

Male-to-male sexual activity is no longer punishable by law in China [340, 341]. The Chinese supreme court has ruled to exclude sodomy as a criminal act in 1957 [102]. The Chinese government abolished the “Hooliganism Law” from the Chinese Criminal Code in 1997 [342, 343], which signifies the decriminalisation of homosexuality in China. Furthermore, the term “homosexuality” was also removed from the list of psychiatric disorders by the Chinese National Psychiatric Association in 2001 [344]. With the increasingly permissible legal environment, the first HIV sentinel surveillance (HSS) site to target MSM was established in Heilongjiang Province in 2002 [345]. Two additional HSS sites for MSM were established in Anhui and Henan Provinces in 2005 [345]. The number of HSS sites further increased to 17 in 2009, covering eleven Chinese provinces [346]. Since then, there was a dramatic 6-fold increase in the number of HSS sites during 2009–2011 [347]. Currently, China hosts 108 HSS sites, monitoring HIV transmission and risk behaviours among MSM in all 31 Chinese provinces except the Tibet Autonomous Region (Figure 2) [14]. Routine epidemiological and behavioural information are collected in annual cross-sectional surveys [348, 349]. Participants are recruited through various methods, including snowball, venue-based, and internet recruitment sampling methods [14, 347]. Despite of this large scale-up of surveillance efforts, the current surveillance coverage remains insufficient to capture the trend of HIV and sexually transmitted infections (STIs) among MSM in many parts of the country [347, 350]. Recently, the central government funding for the HIV/AIDS responses has significantly scaled up from RMB 1.1 billion (~US$ 154.2 million) in 2008 [5] to RMB 3.4 billion (~US$ 497.3 million) in 2010 [351]. However, only US$ 4.4 million in 2008 and US$ 12.7 million in 2010 were set aside for MSM, accounting for only 2-3% of the total funding [148].

HIV testing service is a key component of HIV surveillance [352, 353]. Despite a significant increasing trend of HIV annual testing rate among MSM (from 11.0% in 2003 to 50.4% in 2011) [11], approximately 61.1–87.0% of HIV-infected MSM remain undiagnosed [18, 354]. The low HIV testing rate among Chinese MSM is associated with a number of psychological and structural barriers. The majority of MSM perceive themselves as healthy and with low risk of acquiring HIV [31, 355]. Double social stigma against gay men and HIV patients in China complicate MSM to disclose their sexual orientation and/or HIV positive status [20, 355, 356]. The Chinese Stigma Index Report revealed that 25% of Chinese medical staff had negative and discriminatory attitudes towards people living with HIV (PLHIV) in 2009 [357]. Lacking of trust obstructs the uptake of HIV testing and subsequent medical procedures among MSM [31, 358, 359]. Unawareness of HIV serostatus among HIV-infected MSM may continue to fuel the spread of the virus [358]. In terms of structural barriers, a large proportion of MSM are not aware of the locations of any HIV testing site in their neighbourhood [20, 355, 356], likely due to the lack of outreach of HIV intervention programs for MSM. However, successful roll-out of any of these interventions' programs relies on their ability to protect the identity and privacy of MSM. Due to the anonymous nature of the Internet, a study revealed that the majority of Chinese MSM (84.7%) would prefer receiving HIV/AIDS-related intervention via Internet, instead of receiving the information from the China Centers for Disease Control and Prevention (CDC) (28.4%) and hospitals (22.8%) [360]. This shows that the non-face-to-face Internet-based intervention is a more acceptable approach to Chinese MSM.

## 6. Community Responses to HIV among Chinese MSM

### 6.1. Development of Community-Based Organizations in China

Since early 1990s, several gay men voluntarily joined together and started to advocate for HIV prevention and awareness and knowledge of HIV/AIDS to the gay community [361]. They established the first telephone hotline ‘‘99575 Beijing Tongzhi Hotline” in Beijing, this hotline was served by health educators in order to provide health promotion, counselling services, harm reduction strategies, and safe sex practices to the local MSM community [102]. In 1997, a group of MSM established a community-based program named “Friends” in collaboration of with specialists and professions from public health, sociology, psychology, and legal areas. This program led to the subsequent publication of a bimonthly magazine named “Friend Exchange” in the following year. This magazine provided a comprehensive collection of information on HIV/AIDS, sexual orientation, academic researches, and personal life experiences of homosexual individuals [362–364]. This program gradually expanded and was transformed into the first registered community-based organization (CBO) for gay and lesbians (i.e., the Beijing Gender Health Education Institute) in Beijing in 2002 [102]. The institute provided training program to promote self-acceptance of sexual identity and social justice and provide related psychological counselling to its participants. Furthermore, with the support of the Fifth Round of AIDS Program of Global Fund in China in 2005, this program had been significantly scaled up to promote HIV prevention and AIDS treatment among MSM [365]. The success of this program has led to the Chinese Ministry of Health's decision to explicitly request all level of health departments to initiate health intervention programs among MSM [363]. Consequently, a large number of grassroots CBOs for MSM have been then established in major urban cities. A large cross-sectional study among MSM in 61 cities was conducted in 2009 to understand the geographical disparities of HIV epidemic and risk behaviours among the population [48], which has initiated a strong collaboration between local CDCs and MSM-targeted CBOs [366]. In 2013, China CDC has pledged to provide greater support to CBOs participating in HIV/AIDS prevention activities [352].

### 6.2. The Unique Role of CBOs in HIV Surveillance and Prevention

CBOs play a unique role in confronting the HIV/AIDS epidemic among MSM in China. First, unlike other high-risk populations such as female sex workers and injecting drug users, gay relationships are not illegal in China and hence the Chinese government cannot exert authority over this population. In general, it is difficult for individual MSM to establish a well-trusted relationship with governmental bodies [359]. Due to social discrimination towards homosexuality and people living with HIV (PLHIV), most of Chinese MSM will conceal their homosexuality publicly [103]. Without the mediation of well-trusted CBOs that are representative of the MSM community, it has become apparent that the Chinese government cannot access to this population and conduct an effective epidemic surveillance [367–369]. Second, in comparison with the governmental institutions, CBOs are much less authoritative and more extensively rooted in the MSM community [370]. These organisations are capable of mobilising multiple channels through private entertainment establishments (e.g., gay bars, saunas), public venues (e.g., parks and clubs), and mass communication media (e.g., internet and hotlines) to effectively provide peer-education services, free condom and lubricant distribution, and peer-counselling and promote HIV voluntary counselling and testing (VCT) [371]. Third, CBOs are usually at a better position to engage the government to advocate for changes in health policies, allocation of resources, and rights for their community.

### 6.3. Barriers and Challenges Facing the Development of CBOs

Development of CBOs in MSM community faces several challenges. First, financial restriction obstructs the official registration of CBOs [372]. In particular, establishing a CBO at a national or local level in China requires a minimum of RMB 100,000 (US$ 15,000) and RMB 30,000 (US$ 4,500), respectively [373]. Due to the lack of enabling financial, political, and legal environment [372], very few of them obtain registered status [374]. As a result, they are not eligible to apply for governmental funds and are at risk to be banned by the Chinese government. Currently, the operation and development of MSM-based CBOs are mainly supported by external funding bodies such as Global Fund to Fight AIDS, Tuberculosis and Malaria, which is the largest funding source for Chinese CBOs [375, 376]. With the gradual withdraw of Global Fund and eventual termination in China in 2013, many of these CBOs is expected to dissolve in the absence of replacement funding from the government [377, 378]. Second, CBOs are lacking capacity [379–381]. Most of these organizations remain focusing on the primary intervention activities such as condom distribution and health advocacy but have limited experience on financial management, funding application, project planning, management, organization, and supervision [381]. The quick staff turnover, lack of collaboration with governmental bodies and research institutions also limit its development and expansion in the community [211].

## 7. Scientific Innovations in Responses to HIV among MSM

Several biobehavioural interventions have been shown to have significant impacts in preventing HIV transmission in recent years. First, male circumcision could significantly reduce 50–60% of HIV transmission via penile-vaginal sexual intercourse [382–384]. It is estimated that less than 5% of the total male Chinese population are circumcised [385]. However, 30.7–36.4% of MSM are willing to undergo circumcision [291, 386, 387]. Second, HIV preexposure prophylaxis (PrEP) may reduce the chance of HIV acquisition during sexual intercourse [388]. Studies showed that only 11.2% of MSM in Beijing are aware of PrEP but 67.8% are willing to use PrEP, if it is available in China [389]. An even higher rate of awareness of PrEP (22.1%) is reported among MSM in the Southwest China, where HIV is most prevalent [390].

## 8. Conclusions 

MSM is an emerging highly at risk population for HIV transmission in China. Overlapping homosexual, bisexual, and commercial sexual activities and high-risk drug use behaviours are common among Chinese MSM. Due to the hidden nature of the population and the existing stigma and discrimination associated with HIV infection and homosexuality, Chinese MSM are reluctant to access healthcare and HIV testing services [40]. Hence, it remains a challenge to provide timely diagnosis, care, and treatment to HIV-infected individuals. A substantial scale-up of epidemiological and behavioural surveillance efforts is required. Furthermore, innovative technology-based HIV prevention via mobile apps, Internet, or short message service (SMS) should be promoted to target the unreached MSM subgroups [391]. Over the past decade, both Chinese government and grassroots CBOs have significantly increased their commitment and contribution towards HIV surveillance among MSM. CBOs play an irreplaceable role in the national surveillance effort and its close collaboration with the government is essential for any effective epidemic surveillance and prevention measures among Chinese MSM.
